# Function of *FT* in Flowering Induction in Two *Camellia* Species

**DOI:** 10.3390/plants13060784

**Published:** 2024-03-10

**Authors:** Xiong Wang, Jiyuan Li, Hengfu Yin, Xinlei Li, Weixin Liu, Zhengqi Fan

**Affiliations:** 1 Research Institute of Subtropical Forestry, Chinese Academy of Forestry, Hangzhou 311400, China; liuweixin@caf.ac.cn (X.W.); jiyuan_li@126.com (J.L.); hfyin@sibs.ac.cn (H.Y.); lixinlei2020@163.com (X.L.); lwx060624@163.com (W.L.); 2 College of Forestry, Nanjing Forestry University, Nanjing 210037, China

**Keywords:** *Camellia azalea*, *Camellia japonica*, FT, flowering regulation

## Abstract

*FLOWERING LOCUS T* (*FT*), belonging to the *FT*/*TFL1* gene family, is an important gene regulating the flowering transition and inflorescence architecture during plant development. Given its importance to plant adaptation and crop improvement, *FT* has been extensively studied in related plant research; however, the specific role and underlying molecular mechanisms of *FT* in the continuous flowering of perennial plants remains elusive. Here, we isolated and characterized homologous *FT* genes from two *Camellia* species with different flowering-period phenotypes: *CaFT* was isolated from *Camellia azalea*, a precious species blooming in summer and flowering throughout the year, and *CjFT* was isolated from *C. japonica*, which blooms in winter and spring. The major difference in the genes between the two species was an additional five-amino acid repeat sequence in *C. japonica*. *FT* showed high expression levels in the leaves in both species from January to August, especially in April for *C. japonica* and in May for *C. azalea*. *CaFT* was expressed throughout the year in *C. azalea*, whereas *CjFT* was not expressed from September to December in *C. japonica*. The expression levels of *FT* in the floral buds were generally higher than those in the leaves. Overexpression of *CaFT* and *CjFT* in *Arabidopsis* indicated that both genes can activate downstream genes to promote flowering. Transgenic callus tissue was obtained by introducing the two genes into *C. azalea* through *Agrobacterium*-mediated transformation. Transcriptome and quantitative real-time polymerase chain reaction analyses indicated that both florigen *FT* genes promoted the expression of downstream genes such as *AP1*, *FUL*, and *SEP3*, and slightly up-regulated the expression of upstream genes such as *CO* and *GI*. The above results indicated that *CaFT* and *CjFT* played a role in promoting flowering in both camellia species. The expression pattern of *CaFT* in leaves suggested that, compared to *CjFT, CaFT* may be related to the annual flowering of *C. azalea*.

## 1. Introduction

Various external and internal factors affect the process of plant flowering, such as temperature, light, and hormones. Seven induction pathways of flowering in higher plants have been identified to date, including the vernalization, thermosensitive, photoperiodic, gibberellin (GA), autonomous, floral inhibition, and age pathways [1,2]. *FLOWERING LOCUS T* (*FT*) is an important integration factor involved in plant flowering regulation and is one of the key genes regulating flowering, acting as a florigen [3,4,5,6]. *FT* integrates signals sensed by several pathways, which are then transmitted to downstream flowering development-related genes to ultimately promote plant flowering. Exposure to long-day conditions induces the production of the gene encoding the B-box zinc finger transcription factor CONSTANS (CO) in *Arabidopsis* leaves, which directly acts on the promoter region of *FT* or coordinates with other transcription factors to promote *FT* expression in sieve tube companion cells [7]. FT protein is transported to the shoot apical meristem (SAM) (Liu et al., 2012) [8], where it forms a complex with the transcription factor FLOWERING LOCUS D (FD). The FT/FD protein complex then activates the expression of downstream genes such as *SUPPRESSOR OF OVEREXPRESSION OF CONSTANS1* (*SOC1*) and *APETALA1* (*AP1*) to promote flowering [9].

The function of *FT* in promoting early flowering has been reported for various plant species. Overexpression of *FT* in *Arabidopsis* can cause an extremely early flowering phenotype, while the *ft* mutant exhibits a late-flowering phenotype [10,11]. Expression of the *FT* homologous gene in rice, *HEADINBG DATE 3a* (*Hd3a*), results in the transformation plant producing an extremely early flowering phenotype that is not dependent on the photoperiod [12]. Overexpression of *JcFT* from *Jatropha curcas* in *Arabidopsis* and in *Jatropha* driven by the 35S or *SUC2* promoter also results in an extremely early flowering phenotype, and the flower buds are directly initiated from the transformed calli. The expression of several flowering genes acting downstream of *JcFT*, such as *LFY*, *AP1*, and *SOC1*, is up-regulated in *JcFT*-overexpression transgenic plant lines [13]. Among the *FT* genes *CsFTL1*, *CsFTL2*, and *CsFTL3* from *Chrysanthemum seticuspe*, only the expression of *CsFTL3* is up-regulated under floral-inductive conditions [14]. In ornamental flowers, correlation analysis of three *RoFT* genotypes and flowering-time phenotypes in the mapping population of rose showed that the *RoFT_f* and *RoFT_g* alleles contribute to the early flowering phenotype, while the *RoFT_e* allele contributes to the late-flowering phenotype [15]. The *FT* gene from *Prunus mume* was transformed into the oil-bearing rose, and the earliest three lines flowered within one month, along with a reduced juvenile phase and an increase in lignification of cells in the stem [16].

For the majority of *Camellia* species, the flowering period spans from December to March of the following year. *Camellia japonica* is the model plant for this genus, with the greatest number of varieties. *Camellia azalea* is one of the few plants in the *Camellia* genus that flowers year-round and blooms in summer, thereby serving as an important parent for cultivating continuous-flowering varieties. Thirty hybrids of *C. azalea* consistently tend toward *C. azalea* phenotypes with respect to flower bud formation and the development and blooming period [17]. In rose and strawberry, continuous flowering is controlled by the orthologous gene *KSN* of the *TERMINAL FLOWER 1* (*TFL1*) family [18,19]. In our preliminary research, we found no difference in the sequences of *TFL1* between *C. japonica* and *C. azalea*. Through the transcriptome sequencing of *C. azalea* flower buds, the expression patterns of flowering-related genes during floral bud development were analyzed, and *FT* or *TFL1* genes with decreasing patterns during floral bud development indicated that they might act as floral suppressors, and, thus, as more similar to the Arabidopsis *TFL1* [20]. The expression of *CoFT1*, a homolog of *FT* from *Camellia oleifera*, showed a seasonal change in expression, which was consistent with the formation time of the flower [21,22].

In summary, the differences in the amino acid sequence in non-conservative areas of the *FT* gene may lead to differences in the plant flowering period. To further understand the role and underlying mechanism of *FT* as a key integration factor of flower induction pathways, in this study, we compared the gene sequences, expression patterns, ectopic expression, native transformation, and transcriptomes of *FT* from *C. japonica* and *C. azalea*. These results provide evidence for the regulatory patterns of *FT* genes in two representative *Camellia* species with different flowering patterns.

## 2. Results

### 2.1. Identification and Sequence Analysis of FT Genes in C. japonica and C. azalea

**Figure 1:**
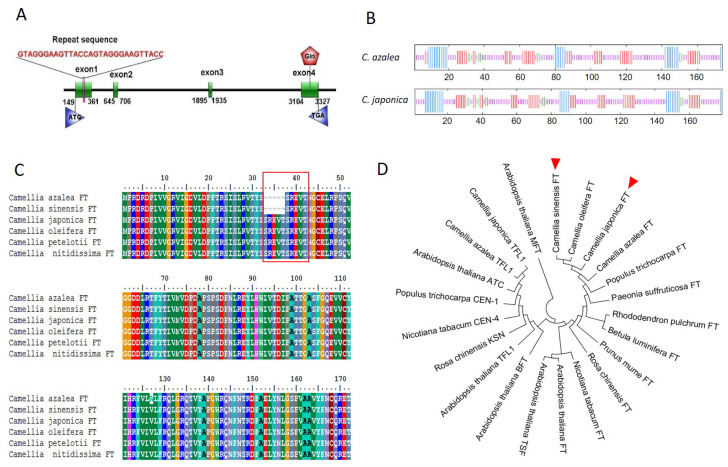
Amino acid alignment and phylogenetic analysis of *CaFT* and *CjFT. (***A**) Predicted gene structure of *CjFT*; an additional 15 bp repeat sequence is found in exon 1 of *CjFT* compared to that of *CaFT*. (**B**) Predicted secondary structures of the CaFT and CjFT proteins, comprising alpha-helixes (blue), random coils (purple), extended strands (red), and beta-turns (green). CaFT and CjFT are mainly composed of α-helixes and random coils, with a greater combination these two structures found at the location of the repeat sequence in CjFT. (**C**) Multiple sequence alignment of CaFT, CjFT, and homologous FT proteins in *Camellia*. The five repeated amino acids are framed in the red box. (**D**) Phylogenetic tree of *CaFT* and *CjFT* showing tight clustering of the *FT* genes of *Camellia*.

Based on the transcriptome data of *C. azalea*, the cDNA sequences of the *FT* gene were cloned from *C. japonica* and *C. azalea,* including open reading frames of 540 bp and 525 bp encoding 179 and 174 amino acids, respectively, which were named *CjFT* and *CaFT*. The DNA sequence of *CjFT*, derived from the genome data of *C. japonica*, was aligned with that of *CjFT* to build the gene structure diagram of *CjFT* (Figure 1A). Four exons and five introns were identified in the DNA sequence of *CjFT*, which contained an additional 15 bp repeat sequence in exon 1 compared to the structure of *CaFT*. Secondary structure analysis showed that the proteins encoded by *CaFT* and *CjFT* were both mainly composed of random coils, extended strands, α-helixes, and β-turns, and the main difference between species was based on a greater combination of random coils and α-helixes at the location of the repeat sequence in *CjFT* (Figure 1B).

Amino acid sequence alignment by BioEdit showed high conservation of the *FT* genes from the two *Camellia* species, with identity of 96.6% owing to the loss of the five-amino-acid repeat in *CaFT*. Multiple sequence alignment showed that the *CaFT* sequence was similar to the *FT* sequence from *C. sinensis*, whereas the *CjFT* sequence was similar to the FT sequences of *C. oleifera*, *C. petelotii*, and *C. nitidissima* (Figure 1C). The phylogenetic tree (Figure 1D) revealed that *FT* genes from the *Camellia* genus clustered together first, followed by those of *Paeonia suffruticosa*, *Betula luminifera, Prunus mume, Rosa chinensis, Nicotiana tabacum,* and *Arabidopsis thaliana*. *FT* genes from these woody plants clustered earlier than those from grass plants. Both genes belonged to the PEBP superfamily. The key amino acid residues Tyr85(Y) and Gln140(Q), differentiating *FT* and *TFL1,* were found in *CaFT* (Tyr84 and Gln139) and in *CjFT* (Tyr89 and Gln144).

### 2.2. Expression Patterns of CaFT and CjFT in the Leaves and Floral Buds

**Figure 2:**
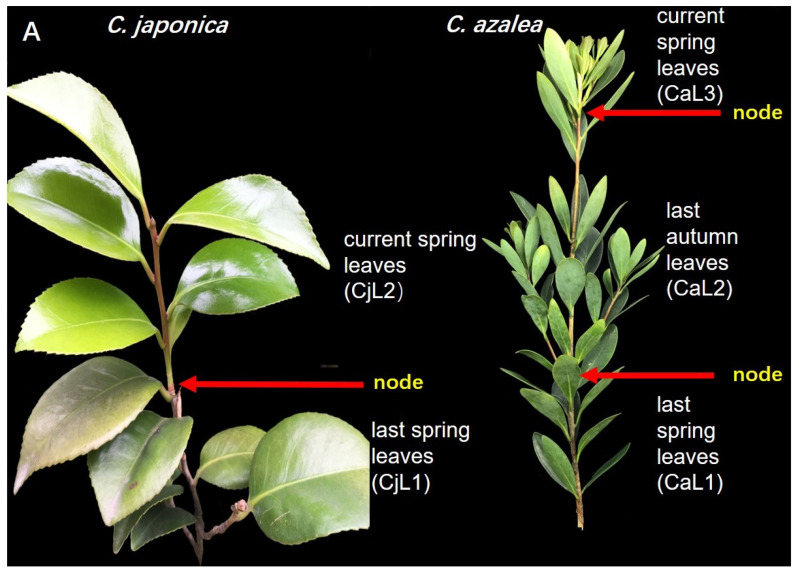

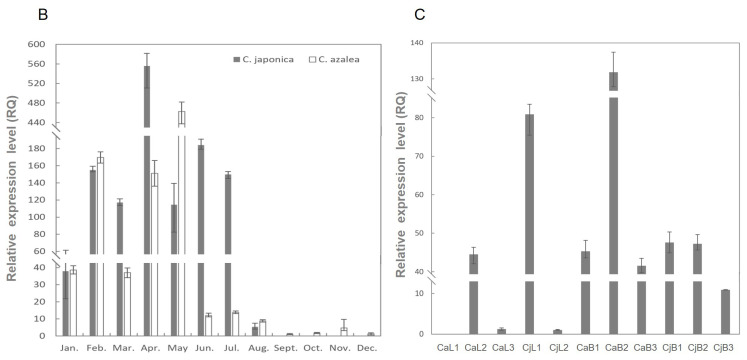
Expression pattern of *FT* in the leaves and floral buds. (**A**) Expression levels of *FT* in the leaves located in different branches, determined by qRT-PCR. (**B**) Comparison of expression levels in the leaves over 12 months between *C. japonica* and *C. azalea*. (**C**) Expression levels of *FT* in the leaves at different locations and in the flower buds at different development stages. CaL1, leaves of the previous spring of *C. azalea*; CaL2, leaves of the previous autumn of *C. azalea*; CaL3, leaves of the current spring of *C. azalea*; CjL1, leaves of the previous spring of *C. japonica*; CjL2, leaves of the current spring of *C. japonica*; CaB1, 2 mm floral buds of *C. azalea*; CaB2, 10 mm floral buds of *C. azalea*; CaB3, 28 mm floral buds of *C. azalea*; CjB1, floral buds of *C. japonica* in June; CjB2, floral buds of *C. japonica* in October; CjB3, floral buds of *C. japonica* in January.

We detected the relative expression levels of *CaFT* and *CjFT* in the leaves at different periods and positions, and of the floral buds at different developmental stages of *C. azalea* and *C. japonica,* using qRT-PCR (Figure 2A). In the monthly leaves of *C. japonica*, the expression of *FT* was detected from January to August, with the highest expression levels observed from February to July. The peak of expression occurred in April, with a level three times higher than that of the second-highest level detected in June. April was also the month during which the largest number of flower buds formed in *C. japonica*. In *C. azalea*, *FT* was expressed throughout the year, with the highest expression levels were found from February to May. The peak of expression was in May, with a 2.7 times higher expression level than that in February, and with the second-highest expression level. Although the expression level sharply decreased as of June, low levels of expression were still detected up to December. The trend of expression level change was highly consistent with the period of flower bud formation in *C. azalea*. Comparing the two species, the expression of *FT* was more concentrated in *C. japonica*, whereas it was relatively scattered across the months in *C. azalea*. Although the expression level was higher in the peak month for *C. japonica*, *C. azalea* had a higher expression level of *FT* than that found in *C. japonica* for 8 months (Figure 2B).

In general, the expression levels of *FT* in the floral buds were higher than those in the leaves. There were no significant differences in the expression levels in the floral buds among different stages, except for a higher expression level found at CaB2 (10 mm floral buds in the rapid growth period of the floral organs) in *C. azalea* and a lower expression level found at CjB3 (when the floral buds were about to bloom after low-temperature induction) in *C. japonica*.

The *C. azalea* plants produce a large number of sprouts twice a year, while in *C. japonica,* they only sprout once. Therefore, there were two types of leaves that could be obtained before the formation of a large number of flower buds in April in *C. japonica*: those from the previous spring and those from the current spring. However, there were three leaf types for *C. azalea* in May: those of the previous spring (CaL1); those of the previous autumn, collected in May (CaL2); and those of the current spring (CaL3). Among the leaves collected from different locations, CaL2 and CjL1 (the leaves of the previous spring of *C. japonica,* collected in April) exhibited relatively higher expression levels. The tender leaves of the current spring showed very low levels of *FT* expression in both species, whereas no *FT* expression was detected in the old leaves of the previous spring of *C. azalea* (Figure 2C).

### 2.3. Introduction of CaFT and CjFT Advanced the Flowering Time of Arabidopsis

**Figure 3:**
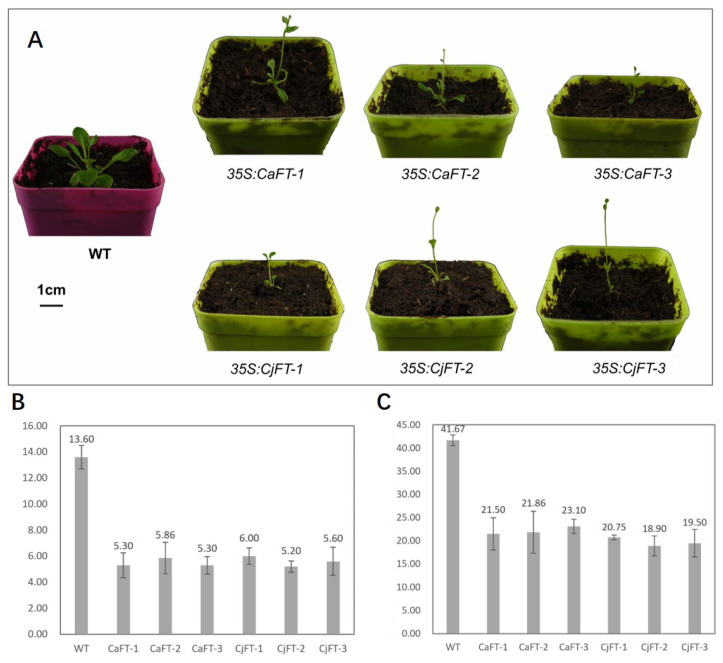
Statistics of flowering time of transgenic plant lines of *CaFT* and *CjFT.* (**A**) The phenotypes of transgenic *Arabidopsis* plants with *CaFT* and *CjFT* and wild-type plants during bolting. Transgenic plants had fewer rosette leaves and were weaker during flowering. (**B**) The numbers of rosette leaves at bolting of transgenic and wild-type plants. (**C**) The days from sowing to bolting of transgenic and wild-type plants.

To identify the function of *CaFT* and *CjFT*, we transformed the genes into the genome of *Arabidopsis* via the inflorescence immersion method in an *Agrobacterium tumefaciens* suspension. Three transgenic plant lines were obtained for both *CaFT* and *CjFT* (Figure 3A). The number of rosette leaves at bolting and the days from sowing to bolting were determined to reflect whether the flowering time of the transgenic plants had changed. There were 13.6 leaves when the wild-type plants bolted, whereas there were only 5.2–6.0 leaves in the transgenic plants at the same stage (Figure 3B). There was no clear difference in the number of leaves between the transgenic plants of *CaFT* and *CjFT*. There was a clear change in the flowering time according to the progression of days from sowing to bolting; the number of flowering days of the transgenic plants was approximately half that of the wild-type plants, and there was a reduction in *CjFT* plants of 1–4 days compared to the number of flowering days of *CaFT* plants (Figure 3C).

**Figure 4:**
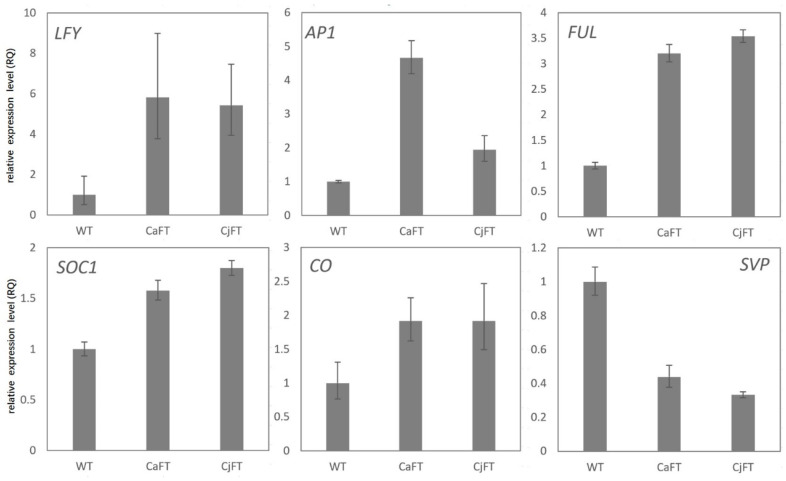
Expression of six genes involved in the flowering pathway in transgenic and wild-type plants. The results of *CaFT*-transgenic, *CjFT*-transgenic, and wild-type (WT) plants are averaged, respectively.

Through qRT-PCR verification, the exogenous *FT* genes were not detected in wild-type plants, whereas they were highly expressed in transgenic plants, and their cycle threshold values were similar to those of the housekeeping gene *ACTIN*. To identify the regulation of exogenous *FT* genes on flowering-related genes in transgenic plants, the expression levels of six genes involved in the flowering pathway were detected. The expressions of *LFY*, *AP1*, *FUL*, *SOC1*, and *CO*, as flowering-promoting factors, were up-regulated, while the expression of *SVP*, as a flowering-inhibiting factor, was down-regulated in the transgenic plants (Figure 4).

### 2.4. Transgenic Calli of CaFT and CjFT Were Successfully Obtained from C. azalea

**Figure 5:**
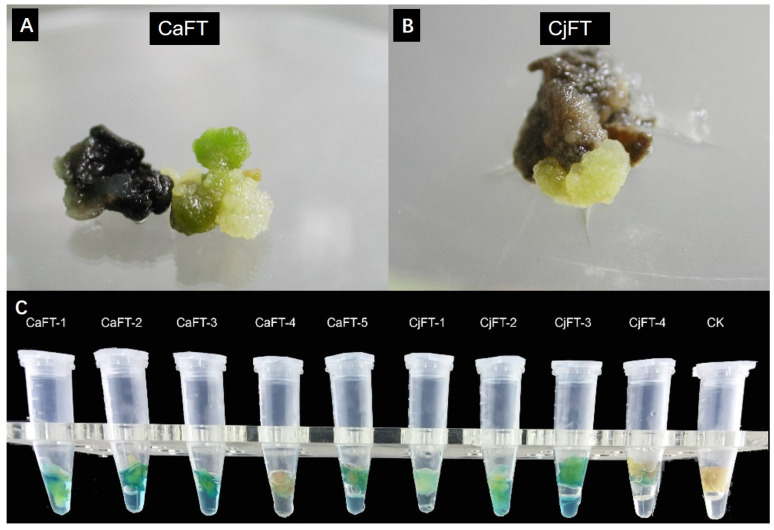
Transgenic callus tissues of *CaFT* and *CjFT* were obtained from *C. azalea.* (**A**) Transgenic callus tissue expressing *CaFT* grew from the selected callus tissue of *C. azalea*. (**B**) Transgenic callus tissue expressing *CjFT* grew from the selected callus tissue of *C. azalea*. (**C**) GUS staining of the callus tissues. CK, non-genetically modified callus tissue with no change; the five *CaFT* calli and four *CjFT* calli show positive GUS staining (blue).

Based on the success of regenerated plants from the stems of *C. azalea*, we attempted to introduce *CaFT* and *CjFT* into the genome of *C. azalea*. After approximately 50 days of selective cultivation with hygromycin, the majority of the infected stems died gradually under the selection pressure, and only a few new calli emerged from the seemingly dead explants. Five transgenic tissue blocks of *CaFT* and four transgenic tissue blocks of *CjFT* were produced from the infected explants (Figure 5A,B). After PCR identification, new calli amplified the target gene, while the control group did not. The proliferative transgenic calli were detected by blue GUS staining (Figure 5C). These results confirmed the presence of both the *CaFT* and *CjFT* genes in the genome of the transformed *C. azalea* callus.

### 2.5. Transcriptome Analysis of the Transgenic Calli of CaFT and CjFT

Transcriptome analysis was carried out to compare the gene expression levels in the calli of transgenic *CaFT*, transgenic *CjFT,* and non-modified plants, named CaFT, CjFT, and NT, respectively. A total of 65.54 Gb of clean data were obtained from nine samples, with no less than 6.14 Gb for a given sample. The Q30 was 94.73% and above. After assembly, we obtained 149,782 transcript sequences and 99,883 unigenes, with expression detected for 147,279 sequences and 97,926 unigenes. The results of the correlation and principal component analyses showed good repeatability between three samples in the same group (Supplementary Figure S1).

The 50,866 unigenes were annotated by comparison with six functional databases (NR, Swiss-Prot, Pfam, COG, GO, and KEGG). The number of annotated unigenes in these databases ranged from 13,523 to 41,055.

**Figure 6:**
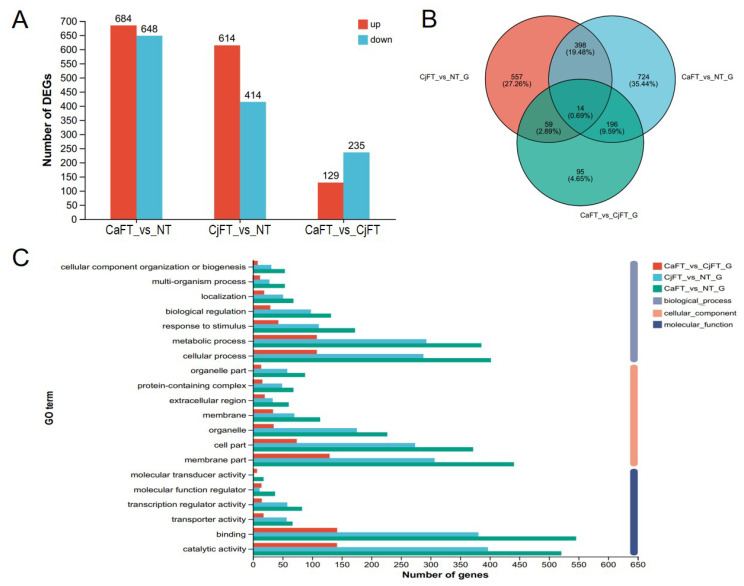
Statistical analysis of differentially expressed genes of the transgenic calli from transcriptome data between the CaFT, CjFT, and NT (non-transformed) groups. (**A**) Expression difference between transgenic CaFT, transgenic CjFT, and NT (non-transformed) calli. (**B**) Venn diagram of the differentially expressed genes (DEGs). (**C**) Gene ontology (GO) classification of gene sets.

Differentially expressed genes (DEGs) were identified according an adjusted *p*-value < 0.05 and fold change ≥2 of the transcripts per million (TPM) value as criteria thresholds using DEseq2. In the gene sets of CaFT vs. NT, CjFT vs. NT, and CaFT vs. CjFT, there were 1332, 1028, and 365 DEGs, including 684, 614, and 129 up-regulated genes and 648, 414, and 235 down-regulated genes, respectively (Figure 6A,B). The number of DEGs showed a large difference between the transgenic and non-transgenic calli, while there was a very minimal difference between the two transgenic calli. Among them, there were 67 up-regulated DEGs with Log 2FC exceeding 5, and 45 down-regulated DEGs with Log2 FC below −5 between the transgenic and non-transgenic calli (Supplementary Figure S2).

GO analysis showed that the DEGs of the three gene sets similarly clustered according to the three categories of molecular function, cellular component, and biological process. Cellular process and metabolic process occupied the top two terms in the biological process category. Membrane part, cell part, and organelle had a higher proportion in the cellular component category. Catalytic activity and binding were the main molecular function terms (Figure 6C).

### 2.6. Differential Regulation of Floral Induction and Floral Organ-Related Genes by FT

**Figure 7:**
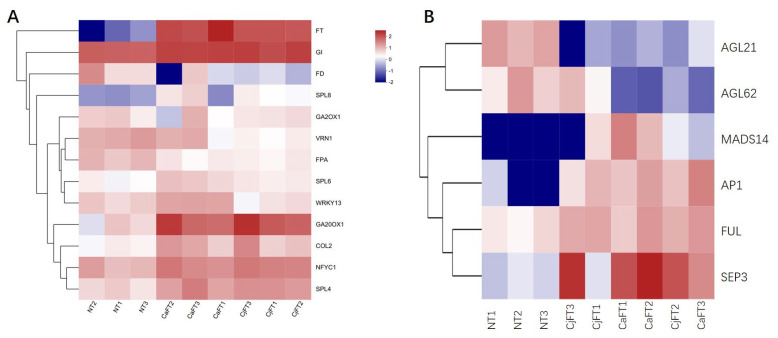
Heat map of the expression levels of genes involved in flowering pathways from the transcriptome data (**A**). The genes of flowering induction pathways; *FT* and its 12 upstream flowering-related genes. (**B**) MADS-box genes participated in the flowering pathway; six MADS-box genes were located downstream of *FT*. The numbers from −2 to 2 represented the changes in Log10- transformed relative TPM value from low to high, and zero mean log10 TPM = 0.

By searching in the six function databases, we found 120 unigenes participating in flowering pathways and 38 unigenes of MADS-box. The expression levels of 13 unigenes of the former and 6 unigenes of the latter showed a significant difference between the transgenic and non-transgenic calli. *CjFT* and *CaFT,* as foreign genes, were barely detected in the NT calli, whereas their respective TPM values reached an average of 61.72 and 114.61 in the CjFT and CaFT transgenic calli. In the upstream regulation pathways of *FT*, some genes related to the photoperiod, autonomous, GA, and vernalization pathways appeared to be up-regulated or down-regulated after insertion of the foreign *FT* genes. Specifically, *GI*, *COL2*, *NFYC1*, *GA20OX1*, *SPL4*, *SPL6*, *SPL8*, *WRKY13*, *FPA*, and *MADS14*, as positive regulatory factors in the flowering induction pathway, were up-regulated, whereas the negative regulatory factors *GA2OX1*, *VRN1*, *AGL21*, and *AGL62* were down-regulated. Some downstream genes of *FT*, such as *AP1*, *FUL*, and *SEP3*, were significantly up-regulated. The expression levels of *AP1* and *SEP3* greatly increased due to the overexpression of *FT*. No significant differences in the expression levels of these genes were found between CjFT and CaFT, although the expression levels of up-regulated genes from CaFT were slightly higher than those from CjFT (Figure 7).

### 2.7. Validation of the Key Genes of Transgenic Calli by qRT-PCR

**Figure 8:**
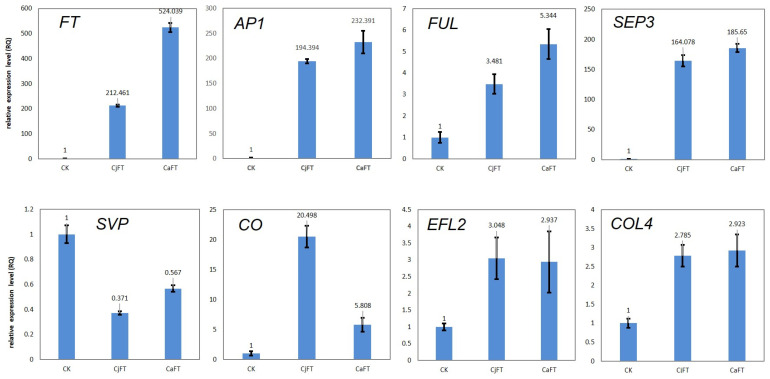
Validation of several flowering-related genes in transgenic calli with *CaFT* and *CjFT* by quantitative real-time polymerase chain reaction.

To verify the reproducibility and reliability of the transcriptome data, we detected the expression of the genes listed above via qRT-PCR. The expression patterns of most of these genes had a similar tendency to that found in the RNA-seq data. The genes located upstream of *FT* did not show significant differences in expression between transgenic and non-transgenic calli, whereas substantial differences in expression were found for the genes downstream of *FT*. In particular, the expression levels of *AP1* and *SEP3* showed a difference of over 150 times, and that of *FUL* showed a difference of over three times. The expression level of *FT* in CaFT was more than double that of CjFT, whereas there were only minor differences in the expression levels of *AP1*, *SEP3,* and *FUL* between CaFT and CjFT (Figure 8).

We also checked the expression levels of some key genes that did not emerge as DEGs in the transcriptome data. Only four genes displayed more than a two-fold difference in expression between transgenic and non-transgenic calli. *SVP* expression, which acts as an inhibitory factor, was down-regulated, whereas the expression of the promoting factors *CO*, *EFL2*, and *COL4* was up-regulated. The expression level of *CO* in CjFT was four times higher than that in CaFT; minimal differences between CaFT and CjFT were found for the expression levels of the other genes (Figure 8).

In general, the results of qRT-PCR were consistent with the transcriptome data with respect to the downstream genes of *FT*. The expression levels of most of the genes evaluated were higher in transgenic calli than in non-transgenic calli, and were only slightly higher in CaFT than in CjFT.

## 3. Discussion

*FT* is well-established to play a significant role in the flowering regulatory mechanism, and its amino acid sequence is highly conserved. Mutations in most residues do not significantly affect the flowering regulation function of *FT* [23]. However, the mutation of any of five key residues (Tyr-85, Glu-109, Trp-138, Gln-140, and Asn-152) would convert the flowering-promotion function of *FT* into the flowering-inhibition function of *TFL1* [23,24,25]. Several *FT* homologous genes in many species are involved in induced flowering, whereas only one member typically plays a major role in this function. For instance, *Hd3a* and *RFT1* in rice regulate the flowering of plants under short-day and long-day conditions, respectively, and *Hd3a* plays a more critical role because rice is a short-day plant [12,26,27]. Only *ZCN8* in *Zea mays* was identified as a flowering-promoting factor, although there are at least eight *FT* homologous genes [28]. Six *FT* genes in soybean can promote flowering in *Arabidopsis* [29,30,31], with *FT2a* identified as a major gene among them [30,31,32]. In kiwifruit, *AcFT2* displays greater flowering activation efficiency than *AcFT1* [33]. Reig et al. (2017) [34] found that *EjFT1* is involved in bud sprouting and leaf development, whereas *EjFT2* might play a role in floral bud induction. We found only one copy of *FT* homologous genes in *C. azalea* and *C. japonica,* respectively, along with the loss of a repetitive five-amino acid sequence of FT in *C. azalea*. The missing amino acid repeat region was located in a non-conserved domain; thus, *CaFT* and *CjFT* could be regarded as homologous genes of *FT* in *Camellia*, although their efficiency likely varies. *C. azalea* shared the same amino acid sequence of FT with *C. sinensis*, but the flowering period was slightly different. *C. sinensis* was flowering from August to May of the following year and blooming in October and November, while *C. azalea* was flowering continuously throughout the year and blooming from June to July. Although it did not bloom continuously throughout the year, *C. sinensis* had a longer flowering period compared to other Camellia species such as *C. japonica*. Although not very close, there seemed to be some connection between the traits and sequences of *FT*. Through their transformation in *Arabidopsis*, both *CaFT* and *CjFT* promoted flowering. The expression levels of flowering integration factors *LFY*, *AP1*, *FUL,* and *SOC1* were up-regulated. This result was consistent with those of *Jatropha* and *rosa* [13,35]. The flowering times of the plants with *CaFT* were slightly earlier than those of plants expressing *CjFT*, which indicated that the promoting effect of *CaFT* on flowering was slightly stronger than that of *CaFT*.

The expression of *FT* in *Arabidopsis thaliana* or *Oryza sativa* responds to the length of sunshine, and *FT* only accumulates under suitable sunshine conditions for flowering. *AtFT* only accumulates under long-day conditions [11], while *Hd3a* can accumulate to a certain level under short-day conditions suitable for its flowering [12]. In the present study, we found that *CjFT* accumulated in long-day conditions and did not accumulate in short-day conditions. This result was in concert with the characterization of *C. japonica* as a long-day plant. However, the photoperiodic response type of *C. azalea* is unclear. Although the expression level of *CaFT* was high during long-day conditions, a low level of expression was also detected under short-day conditions. As to our result, among the leave at different developmental stages, *FT* was mainly expressed in healthy, mature leaves. The expression levels of *FT* in young and overly aged leaves were both very low. The concentrated expression pattern of *CjFT* was consistent with the concentrated formation of the flower bud of *C. japonica*, whereas the expression pattern of *CaFT* was related to the annual flowering of *C. azalea.* The high expression level of *CjFT* in June and July and in the floral buds may be related to the morphogenesis of flower organs, as *FT* has previously been shown to participate in the process of flower bud development [36].

Overexpression of a target gene in the native plant can help to verify its function. However, the introduction of a foreign gene and the regeneration of transgenic plants have posed technical challenges in *Camellia*. In the past two decades, transformed callus tissues have been obtained in tea plants [37,38], whereas transgenic regeneration plants have never been obtained. For the first time, we obtained callus tissues from *C. azalea* transformed with *CaFT* and *CjFT*, which represents a significant step forward in breaking the technical bottlenecks of establishing a genetic transformation system for ornamental *Camellia*.

We analyzed the transcriptome of the transgenic calli with *CaFT* and *CjFT* along with the non-transgenic calli. The number of DEGs in transgenic calli was far higher than that in non-transgenic calli, which indicated that the overexpression of *CaFT* and *CjFT* in *C. azalea* caused a series of changes in the expression of related genes. The downstream genes of *FT* were directly affected, and the significantly up-regulated genes detected included the MADS-box genes *AP1*, *FUL*, and *SEP3*. *AP1* has a dual function as a determining gene in the floral meristem tissue and in the development of the first and second rounds of the floral organs. *AP1* is activated by *LFY* and *FCA*, and is also directly activated by the complex of the FT and FD proteins [10,11,39,40]. In our study, *AP1* expression was significantly up-regulated, whereas the expression of *LFY* could not be detected, indicating that *AP1* can be directly regulated by *FT* without *LFY*. The accumulation of *FT* also triggered the expression of *FUL* in the apical meristem, thereby promoting formation of the inflorescence meristem and further promoting the transformation of the floral meristem. Our results confirmed the existence of the *FUL* pathway. As an E-class gene, *SEP3* controls the formation of the sepals, which was confirmed to be activated in the transgenic calli. In line with these findings, a previous study in three rose cultivars genetically transformed by the overexpression of *RoFT* showed that the genes directly regulated by *RoFT* (*RoSOC, RoLFY, RoAP1*) and genes related to determining the identity of the floral organs (*RoAP2, RoAG, RoFUL, RoAP3*) were all up-regulated [35]. Therefore, the regulatory effect of the *FT* gene on downstream genes in *C. azalea* was validated, despite only successfully obtaining the transgenic callus tissue.

In addition to these MADS-box genes, some genes involved in the photoperiodic pathway were also found to be slightly up-regulated, while some flowering-inhibitory factors involved in the vernalization and autonomous pathways were slightly down-regulated after transgene expression. Other genes involved in the GA and age pathways also showed a change in expression. These results imply that the overexpression of *FT* originating from both *C. azalea* and *C. japonica* strongly stimulated flowering induction in the *C. azalea* callus tissue.

**Figure 9:**
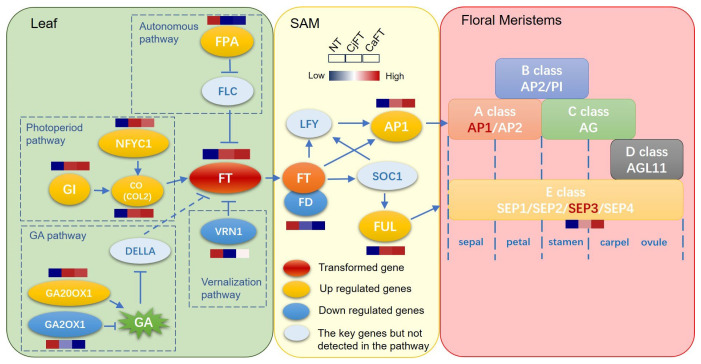
Mechanistic model of the differential regulation of flowering genes by *CaFT* and *CjFT* in transgenic calli. Due to over-expression of *CaFT* and *CjFT* in calli, the flowering-promoting factors *GI*, *NFYC1*, *CO,* and *GA20OX1* were all up-regulated, while the inhibitory factors *GA2OX1* and *VRN1* were all down-regulated. Flowering integration factors *AP1* and *FUL,* located downstream of *FT*, and especially *SEP3* in the ABCDE model, were activated. The flowering regulation effect of *CaFT* was slightly better than that of *CjFT*.

Since *FT* is linked to floral induction and floral organ differentiation, we constructed a regulation model of *FT* (Figure 9) according to the floral induction pathway, the ABCDE model of floral organ development, and the expression of the key genes among NT, CaFT, and CjFT calli. The genes that were validated from the transcriptome data were included in the model. In the leaves, seven genes of four floral induction pathways were influenced by the expression of exogenous *FT* genes. The flowering-promoting factors were all up-regulated, while the inhibitory factors were all down-regulated, except for *FPA*. There was no regularity in the slight difference in upstream gene expression between CaFT and CjFT. Exogenous *FT* genes had a slight regulatory effect on downstream genes acting after the transportation of FT to the SAM. The expression levels of *AP1* and *FUL* were significantly increased, and this effect was slightly higher for *CaFT* than for *CjFT*. The expression of these two genes further promoted the expression of *SEP3* in the floral meristems. Similarly, *CaFT* appeared to be more capable of promoting the expression of *SEP3* than *CjFT*. Therefore, the flowering regulation effect of *CaFT* was slightly better than that of *CjFT*, although both genes had strong promotion effects on flowering according to transgenic callus verification (Figure 9).

The function of *FT* in promoting plant flowering has been confirmed in many plants, including the functional differentiation of different family members of *FT* in the same species [41]. However, few studies have compared the efficiency of *FT* genes from different species in promoting early flowering. We confirmed that the FT protein from *C. azalea* was missing a sequence of five repetitive amino acids found in the FT from *C. japonica*. Slight differences were found in the *Arabidopsis thaliana* and callus tissues of *C. azalea* transformed with *CaFT* and *CjFT*. In general, the promoting effect of *CaFT* on flowering was slightly better than that of *CjFT*. Our results suggest that *CaFT* may be involved in the molecular regulation of the flowering of *C. azalea* throughout the four seasons; however, more direct evidence is needed to determine its specific regulation mechanism.

## 4. Materials and Methods

### 4.1. Plant Materials and Growth Conditions

The plant materials used in this study were collected from *C. azalea* and *C. japonica* with distinct florescence traits. *Camellia azalea* Wei, which is considered a precious species because of its ability to flower year-round with blooming in mid-summer, and *Camellia japonica* L., a model species in the *Camellia* genus that blooms in spring, were grown in the experimental fields at the Research Institute of Subtropical Forestry located in Hangzhou, Zhejiang, China (30° 03′ N, 119° 57′ E), under natural light conditions.

To study the time course of *FT* gene expression in the leaves and floral buds of the two species, we sampled the 12-month leaves collected from January to December, the leaves collected at three locations in *C. azalea* and two locations in *C. japonica*, and the floral buds at three development stages from each species.

### 4.2. Isolation of FT Genes

Total RNAs were isolated from the floral buds and leaves using an RNAprep Pure Extraction Kit (DP441, TIANGEN Biochemical Technology, China), and were reversed-transcribed to cDNA according to the instructions of PrimeScript Ⅱ 1st Strand cDNA Synthesis Kit (6210, TaKaRa, Maebashi, Japan). A pair of specific primers (FTFP: 5′-AGTAGGATTAGATTGGGTTGTG-3′; FTRP: 5′-GTGGTTTCAACTTTCAGGCA-3′) was designed by Primer 3 (https://www.primer3plus.com/index.html, accessed on 11 April 2019) according to the transcriptome data.

Polymerase chain reaction (PCR) was performed in a 50 μL reaction mixture with 100 ng cDNA, 25 μL PrimerSTAR Max Premix (2×), and 2 μL of the specific primers (10 μM). The cycling conditions consisted of an initial denaturing step at 94 °C for 5 min, followed by 35 cycles at 94 °C for 30 s, 54 °C for 30 s, and 72 °C for 1 min, and a final elongation step at 72 °C for 10 min. The PCR was conducted in an ABI Veriti thermal controller with 96 wells (Thermo Fisher Scientific, Waltham, MA, USA). PCR products were separated via electrophoresis on 1.0% agarose gels in 1× TAE buffer and visualized under ultraviolet light after staining with Goldview. The PCR products were purified and recovered using an agarose gel DNA purification and recovery kit (Takara, Japan). The recovered fragments were cloned into the pMD20-T vector (Takara) and finally transformed into *Escherichia coli* DH5α competent cells. The positive colonies were validated by PCR and then sequenced at Tsingke Biotechnology Co., Ltd. (Hangzhou, China).

### 4.3. Sequence Alignment and Phylogenetic Analysis

Sequence alignment was carried out using BioEdit and National Center for Biotechnology Information (NCBI) Blast (https://blast.ncbi.nlm.nih.gov/Blast.cgi, accessed on 6 May 2019) tools [42]. SIM4 (https://pbil.univ-lyon1.fr/members/duret/cours/inserm210604/exercise4/sim4.html, accessed on 6 May 2019) was used to align cDNA and genomic DNA to construct the gene structure diagram. The Expasy-Translate tool (https://web.expasy.org/translate/, accessed on 7 May 2019) was used to translate the nucleotides to amino acid sequences, and SOPMA (https://npsa-prabi.ibcp.fr/cgi-bin/npsa_automat.pl?page=/NPSA/npsa_sopma.html, accessed on 6 May 2019) was used to predict the protein secondary structure. Amino acid sequence alignment was performed using DNAMAN 6.0.40 software, and the phylogenetic tree was constructed with MEGA 6.0 software.

### 4.4. Transformation of FT Genes to Arabidopsis thaliana and C. azalea

The pMD20-T included *FT* genes and plant expression vector pCAMBIA1301 were digested by restriction enzymes (KpnⅠ and XbaI). The target gene bands of the former and the linear band of the latter were then recovered and linked. The recombinant plasmids were imported to *Agrobacterium tumefaciens* EHA105 using a freeze–thaw method.

The inflorescences of *Arabidopsis thaliana* were immersed in the suspension of the engineered strains harboring *FT* genes. The seedlings with hygromycin resistance were identified by PCR and real-time PCR, and the numbers of rosette leaves and the culturing days when the plants bolted were recorded.

The stems of sterile seedlings of *C. azalea* were used for the transformation of *FT* genes by means of the *Agrobacterium*-mediated transformation method. The plant materials regenerated on the selective culture medium were identified using PCR, real-time PCR, and β-glucuronidase (GUS) staining.

### 4.5. Transcriptome Sequencing and Analysis of the Transgenic Callus of C. azalea

The transcriptome sequencing of nine samples of the transgenic callus with *FT* genes (CaFT-1, CaFT-2, CaFT-3, CjFT-1, CjFT-2, CjFT-3) and the non-transgenic callus was carried out by Shanghai Majorbio Bio-pharm Technology Co., Ltd. (Shanghai, China). Sequencing libraries were generated using an llumina Truseq^TM^ RNA sample prep Kit (Illumina, San Diego, CA, USA). RNA sequencing (RNA-seq) was performed using the Illumina Novaseq 6000 platform (Illumina, San Diego, CA, USA).

Clean data were obtained by eliminating adapter reads and low-quality reads from the raw data. The Q20, Q30, and GC values were then calculated to assess the quality of the clean data. Trinity software (v2.6.6) was used for the de novo assembly of clean reads, with min-kmer-cov set to 2 and default parameters used for the other options. Functional annotation of the transcripts was performed based on the following databases: NCBI non-redundant protein sequences (NR), Swiss-Prot (a manually annotated and reviewed protein sequence database), Protein family (Pfam), Clusters of Orthologous Groups of proteins (KOG/COG), Gene Ontology (GO), and Kyoto Encyclopedia of Genes and Genomes (KEGG). Plant transcription factors were predicted using iTAK 18. 12 software and the Plant Transcription Factor Database (http://planttfdb.gao-lab.org/index.php, accessed on 27 October 2022). Coding sequence prediction was performed using the NR, Swiss-Prot, and TransDecoder (3.0.1) databases. Transcript abundance was evaluated using RSEM and is presented in units of transcripts per kilobase of exon model per million mapped reads (TPM). Pearson correlations between biological replicates and the heatmaps were calculated using the online tool of Majorbio Cloud Platform (https://cloud.majorbio.com/page/tools/, accessed on 23 February 2024), with an imported Log10-transformed relative TPM value with Euclidean distance. 

### 4.6. Quantitative Real-Time PCR (qRT-PCR) Analysis

The *GADPH* and *ACTIN* genes were selected as the reference genes for qRT-PCR analysis in *Camellia* and *Arabidopsis*, respectively. A PrimeScript RT reagent kit with gDNA Eraser (RR047, TaKaRa) was used to synthesize the first-strand cDNA. The qPCR system was established using SYBR Prime Ex Tap Ⅱ (Tli RNaseH Plus, RR420, TaKaRa). The reaction was performed on a QuantStudio^®^ 7 Flex system (Applied Biosystems, Foster City, CA, USA) under the following conditions: pre-denaturation at 95 °C for 30 s; 40 cycles of 98 °C for 5 s and 60 °C for 30 s; and 95 °C 15 s, 60 °C for 1 min, and 95 °C for 15 s. The relative expression levels of *FT* and other related genes were measured in different tissues and in different development periods using the 2^−ΔΔCT^ method [43]. The primer sequences are listed in Table 1.

## Figures and Tables

**Table 1:** Sequences of primers for quantitative real-time polymerase chain reaction.

Species	Target Gene	Forward Primers (5′ to 3′)	Reverse Primers (5′ to 3′)
*C. azalea* and *C. japonica*	*GAPDH* *FT* *AP1* *FUL* *SEP3* *SVP* *CO* *EFL2* *COL4*	CTGTCGATGTCTCAGTGGTTGAC ATGACCTTAGGACCTTCTACACTC CAGTCTCAATGGGAGCAGCA TCAAAGTGTCGCCAGTGGAA TTTCCGTTCTCTGCGATGCT CATGAATGAAATTGCCACCCT ATCGGAAGCTGATGGGTTCT CTGGGCTTGGAAATGGAGGT GTTGTTCCAGACCACAACGC	TGATCTCATCATAGGAAGCCTTCTT CATAACAAACCACCTCTTGCC AGCATCCAAGGTCACAAGCA GATGAAGGATGCCACCACCA GCTGTTTAGGGGGCCAAGAT AACATTAGCCACTCCCACTC ACAACCACGAACCAGACTCG GGTCAACCACCCTCCTGATG CTCGCTTCTCTGTCGATCCC
*Arabidopsis*	*ACTIN* *LFY* *AP1* *FUL* *SOC1* *CO* *SVP*	GGTAACATTGTGCTCAGTGGTGG CAGCAGCAGAGACGGAGAAA AGCAGTGGGATCAGCAGAAC AAACGGGTCAGCAAGAAGGA GCTCTCAGTGCTTTGTGATGC GGCTCCTCAGGGACTCACTA AACTCTCCGTTCTCTGCGAC	AACGACCTTAATCTTCATGCTGC CCTTGGTGGGGCATTTTTCG CGGGTTCAAGAGTCAGTTCGA TCCCCCAACTCTCTCCACAA CGGTTTGGTGCTGACTCGAT CCTGCTGCGTTATGGGAAGA GGGCGTGATCACTGTTCTCA

## Data Availability

The data presented in this study are available on request from the corresponding author. The data are not publicly available due to privacy restrictions.

## Supplementary Materials

The following supporting information can be downloaded at: https://www.mdpi.com/article/10.3390/plants13060784/s1, Figure S1: The correlation analysis and principal component analysis between the samples. (A) Heat map of correlation. (B) The principal component analysis between the samples. Figure S2: Heat map of 67 DEGs with Log2 FC exceeding 5 and 45 ones less than -5 between CaFT/CjFT and NT.
