# Supplementary material for: Function of FT in Flowering Induction in Two Camellia Species
Source: Plants (Basel). 2024 Mar 10;13(6):784. doi: 10.3390/plants13060784 (PMC10975465; doi:10.3390/plants13060784)
Supplement: Supplementary file 1 [file plants-13-00784-s001.zip › plants-2886489-supplementary.pdf]

plants

MDPI

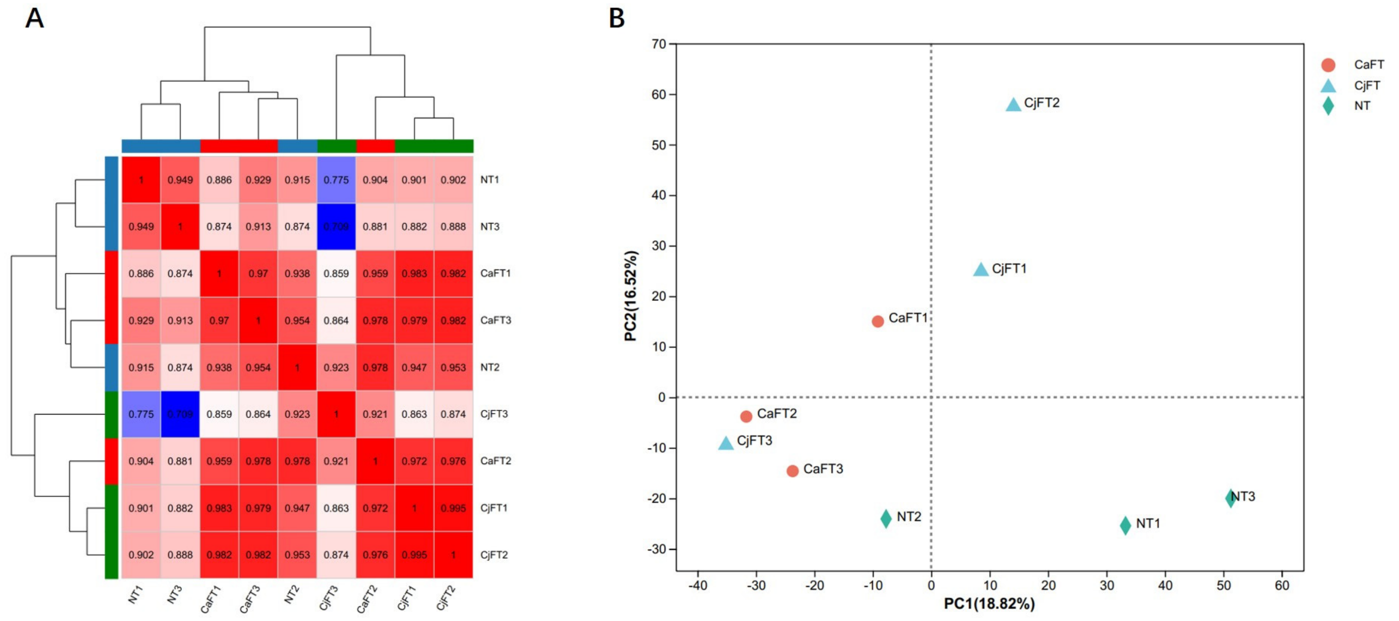

Supplementary Figure 1. The correlation analysis and principal component analysis between the samples A. Heat map of correlation**. B.** The principal component analysis between the samples.

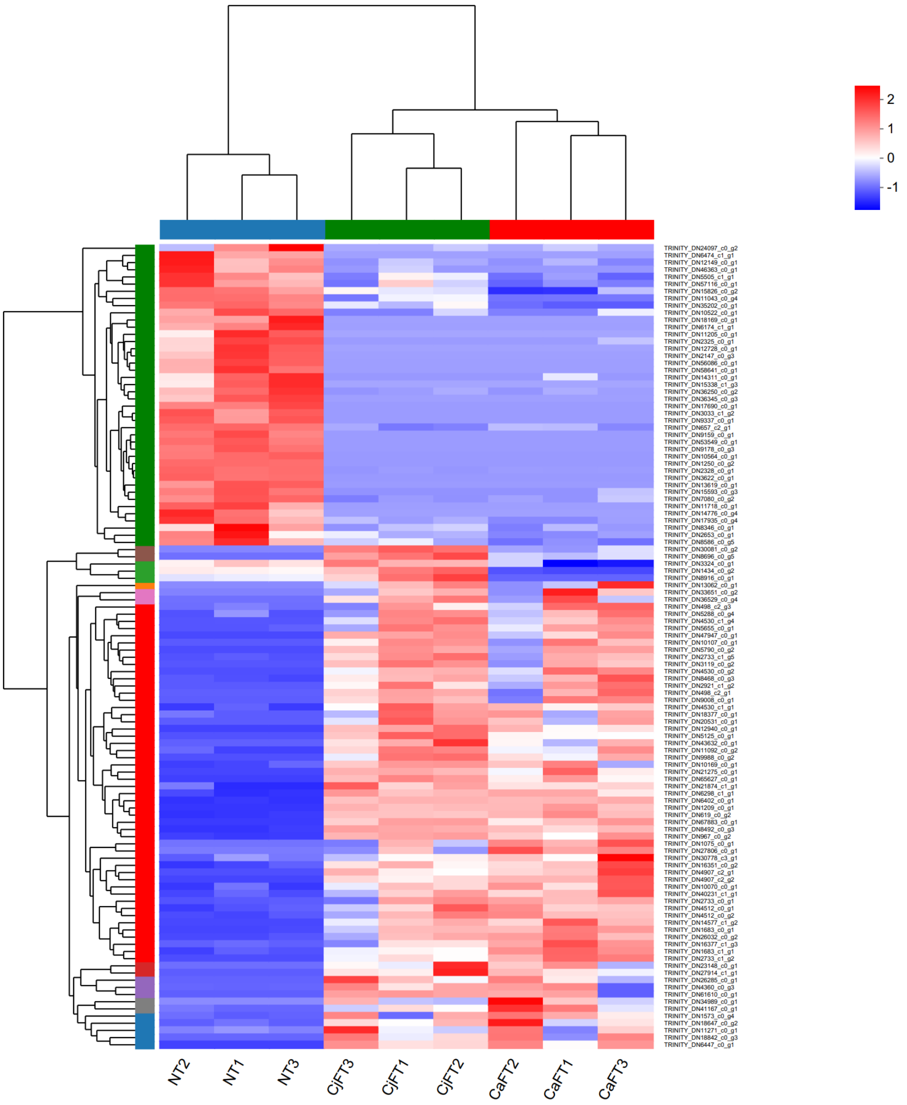

Supplementary Figure 2. Heat map of 67 DEGs with Log2 FC exceeding 5 and 45 ones less than -5 between CaFT/CjFT and NT
